# Prognostic Value of PIK3R4 Expression and Its Correlation with Immune Cell Infiltration in the Diffuse Large B-cell Lymphoma Microenvironment

**DOI:** 10.7150/jca.86681

**Published:** 2023-08-06

**Authors:** Jie Zou, Guangxin Ma, Fei Lu, Jie Li, Chunyan Ji

**Affiliations:** 1 Hematology and Oncology Unit, Department of Geriatrics, Qilu Hospital, Shandong University, Jinan, China.; 2 Department of Hematology, Qilu Hospital, Shandong University, Jinan, China.

**Keywords:** PIK3R4, immune infiltration, DLBCL, biomarker, prognostic indicator

## Abstract

**Background:** As a regulatory unit of class III phosphoinositide 3-kinase (PI3K), PIK3R4 is an important molecule involved in several malignant tumours, but the role and molecular mechanism of PIK3R4 in diffuse large B-cell lymphoma (DLBCL) is still unclear.

**Methods:** Multiple bioinformatics analyses were used to investigate the role and potential mechanisms of PIK3R4 in DLBCL. Quantitative real-time polymerase chain reaction (qRT‒PCR) was performed to determine the expression of PIK3R4 in 80 DLBCL patients, and the survival time of DLBCL patients grouped according to PIK3R4 mRNA expression was compared.

**Results:** PIK3R4 is up-regulated in several malignant tumours, including DLBCL. Bioinformatics analyses revealed that PIK3R4 exhibits prognostic value in DLBCL patients, and the upregulation of this gene in DLBCL samples was subsequently validated. In the functional category, GO analysis revealed that PIK3R4-related genes are enriched in ribosomal RNA metabolic process, the DNA damage response, mitochondrial gene expression, and nucleoside metabolic process. KEGG pathway analysis showed the enrichment of PIK3R4-related genes in the ribosome, oxidative phosphorylation, proteasome, and cellular senescence pathways. More importantly, the expression of PIK3R4 in DLBCL was correlated with the immune cell content in the cancer microenvironment, CD8(+) T-cell and neutrophil infiltration and the levels of several immune checkpoint molecules, including BTN3A2, BTN3A1, PRF1, CXCL9, PDCD1, and TIGIT.

**Conclusion:** Our study demonstrated that PIK3R4, as a novel immune microenvironment-related gene, may represent an important diagnostic, prognostic, or therapeutic biomarker in DLBCL patients.

## Introduction

Diffuse large B-cell lymphoma (DLBCL) is the most common type of aggressive non-Hodgkin lymphoma, and the genetic alterations differ in different stages of the disease and at different stages of treatment 1, 2. In the past two decades, R-CHOP (rituximab, cyclophosphamide, doxorubicin, vincristine, and prednisone) immunochemotherapy has been the standard first-line treatment for DLBCL and has significantly improved patient overall survival; 50%-70% of patients are cured using this approach 3. However, approximately 40% of patients ultimately present with relapsed or refractory disease, and the efficacy of salvage options for refractory/relapsed DLBCL is diminished 4. Obtaining a better understanding of the mechanisms relevant to DLBCL development and immunochemotherapy sensitivity will contribute to identifying new therapeutic targets and prognostic biomarkers for disease diagnosis and treatment 5.

The phosphoinositide-3-kinase regulatory subunit 4 (PIK3R4) gene, located on chromosome 3q22.1, is the regulatory unit of class III phosphoinositide 3-kinase (PI3K). PIK3R4 participates in the formation of the associated protein complex PIK3C3-BECN1-PIK3R4, which is critical for phosphatidylinositol-3-phosphate (PtdIns3P) generation in the initiation stage of autophagy 6, 7. As a cellular defence response to stress conditions, autophagy is crucial for cell homeostasis maintenance. The autophagy process is precisely modulated by variable signalling molecules and includes three relatively independent processes: autophagy induction, formation of autophagic bodies and degradation of mature bodies. Previous studies have reported autophagy activation in the clinical behaviour of several human cancer formations 8, 9. Notably, PIK3R4 is an independent marker of prognosis in chronic lymphocytic leukaemia (CLL), and its upregulation is associated with more aggressive disease 10. PIK3R4 was identified as an important molecule in the process of oesophageal squamous cell carcinoma (ESCC) metastasis 11. Mutations in PIK3R4 are also observed in thymic carcinoma, papillary thyroid carcinoma and metastatic melanoma 12-14. Until now, the molecular mechanisms and clinical significance of PIK3R4 in DLBCL patients have remained obscure.

Our study aims to examine the association between the expression of PIK3R4 and determine the clinical value in DLBCL patients. Multiple bioinformatics analyses were used to extract and analyse datasets from The Cancer Genome Atlas (TCGA), Genotype-Tissue Expression (GTEx), and the Gene Expression Omnibus (GEO) database to investigate the potential oncogenic mechanisms, including correlations between PIK3R4 and the prognosis, immunity and immune checkpoints, of PIK3R4 in DLBCL. Gene Ontology (GO) and Kyoto Encyclopedia of Genes and Genomes (KEGG) pathway enrichment analyses were performed to investigate the potential functions of PIK3R4. In addition, the correlation between the PIK3R4 level and immune infiltration cell-related markers in the tumour microenvironment of DLBCL was determined. More importantly, the expression of PIK3R4 in DLBCL clinical samples was further assessed, and the survival time of patients grouped according to the mRNA expression level of PIK3R4 was compared.

## Materials and Methods

### Study subjects and clinical specimens

**Figure 1:**
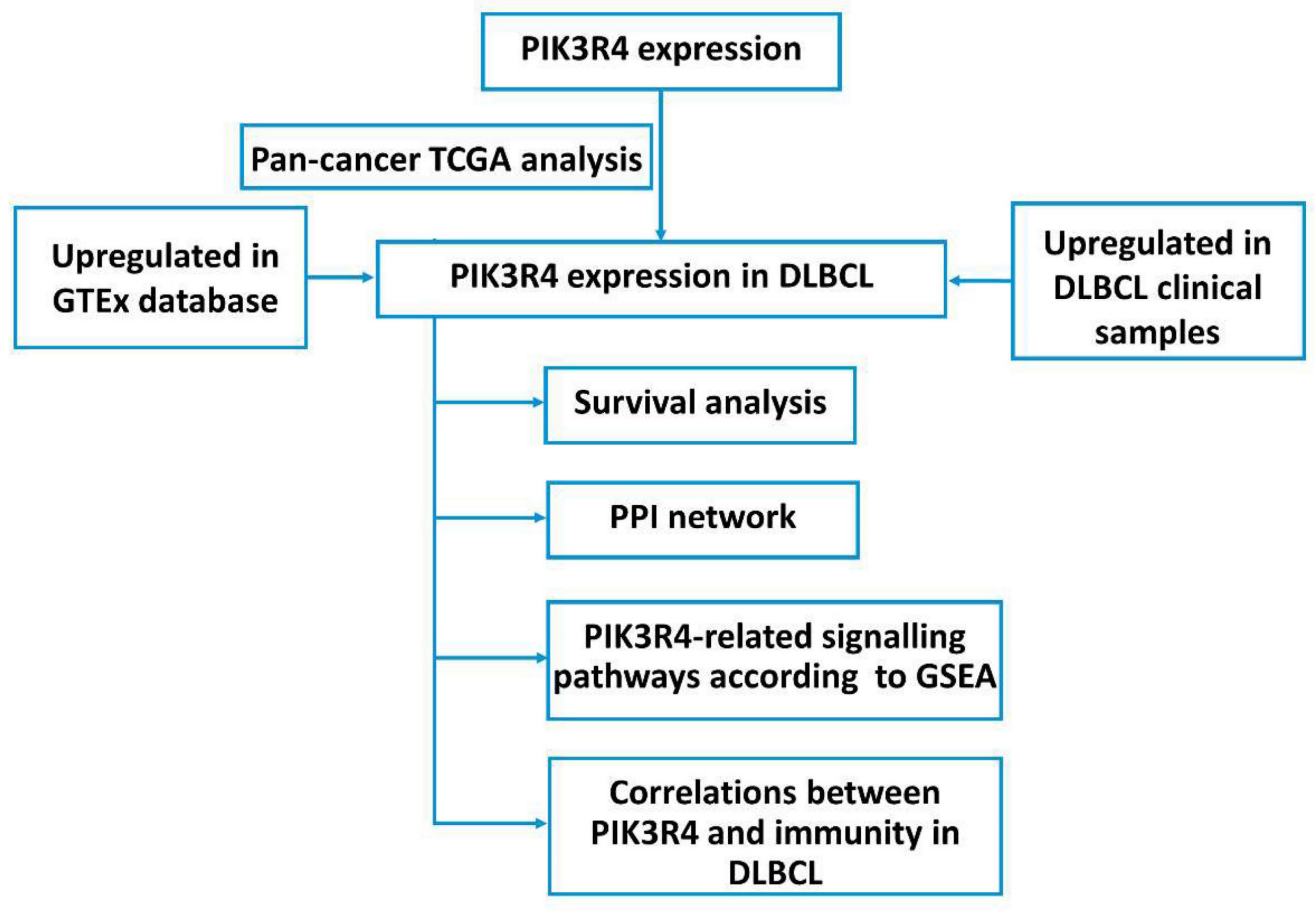
Illustration of the Present Study Workflow.

The general strategy for the present study is shown in Figure 1. In this study, a total of 80 DLBCL tissues and 40 lymphadenitis tissues were obtained from patients at Qilu Hospital between 2016 and 2019. The inclusion criteria were as follows: a) newly diagnosed DLBCL patients, b) ≥18 years old, c) life expectancy > 3 months, and d) diagnosis of DLBCL confirmed by two pathologists according to the 2017 World Health Organization (WHO) classification system of lymphoid neoplasms. The exclusion criteria were the use of chemotherapy or radiotherapy before surgery and a history of human immunodeficiency virus (HIV) or active hepatitis C virus (HCV) infection. The clinical and pathological features of the patients were obtained by reviewing the medical records. All experiments were approved by the Ethics Committee of Qilu Hospital, Shandong University (Jinan, China), and informed consent was obtained from all patients.

### Quantitative real-time PCR (qRT‒PCR)

TRIzol (Invitrogen) was used to extract RNA from tissues, and a cDNA synthesis kit (Invitrogen) was used to prepare cDNA. Then qRT‒PCR was performed using a LightCycler 2.0 Instrument (Roche, Penzberg, Germany) with SYBR Green PCR Master Mix (Toyobo, Osaka, Japan). Samples were run in triplicate and amplified in a 20 μl reaction according to the manufacturer's experimental protocol. The PIK3R4 level in DLBCL samples were separated into "High level" and "Low level" based the qRT-PCR results, and β-Actin expression was used for normalization purposes.

### GEPIA2 dataset analysis

As a gene expression profiling and interactive analysis web server for cancer and normal samples, GEPIA2 (http://gepia2.cancer-pku.cn/#index) provides important customizable and interactive analysis, including profiling plotting, differential expression analysis, correlation analysis, similar gene detection, patient survival analysis, and dimensionality reduction analysis, based on TCGA and GTEx data 15. In our study, GEPIA2 was used to analyse the difference in PIK3R4 expression between normal tissues and tumour tissues in 33 different types of cancer. We compared the expression patterns of PIK3R4 in three tumour types: DLBCL, pancreatic adenocarcinoma (PAAD), and thymoma (THYM). Moreover, GEPIA2 was used for survival curve analysis, including overall survival (OS) curve and disease-free survival (DFS) curve analysis.

### Expression analysis of PIK3R4 in DLBCL based on GEO datasets

Gene expression profile datasets of DLBCL tissues and noncancerous tissues, GSE56315 and GSE32018, were obtained from the GEO database (http://www.ncbi.nih.gov/geo) 16. The expression of PIK3R4 in DLBCL tissues and normal tissues was obtained from GEO datasets and then analyzed. An unpaired t test was used to compare the difference between two groups, and a two-tailed p value of < 0.05 was considered to indicate statistical significance.

### Integrated Protein‒Protein Interaction Analysis of PIK3R4

To illustrate the function of PIK3R4 in DLBCL tumorigenesis and progression, we constructed an integrated protein‒protein interaction (PPI) network with the help of the STRING (https://string-db.org/) website 17 to explore the potential relationships between PIK3R4 and other genes. Genes with a score >0.4 were screened based upon the interaction data in the STRING database.

### Functional and pathway enrichment analysis

We investigated the functional roles and pathway signalling relevance of PIK3R4 in DLBCL. GO and KEGG pathway enrichment analyses were performed via the “clusterProfiler” package 18 and visualized using the “ggplot2” package in R. The GO categories included biological process (BP), molecular function (MF), and cellular component (CC). KEGG analysis defined the pathways associated with PIK3R4 function and co-expressed genes.

### Gene Set Enrichment Analysis

Gene set enrichment analysis (GSEA) was conducted using the online tool LinkedOmics (http://www.linkedomics.org/admin.php). The LinkedOmics database contains multiomics data and clinical data for 32 cancer types and a total of 11158 patients from the TCGA project 19. The differential expressed genes related to PIK3R4 were screened from the TCGA DLBC cohort through the LinkFinder module in the database, and the correlation of results was tested by the Pearson correlation coefficient and presented in a volcano plot and heatmaps. The functional module of LinkedOmics performs analysis of biological process GO and KEGG pathways by GSEA in the LinkInterpreter module.

### Association between tumour microenvironment features and PIK3R4 expression

The TIMER2.0 web server (http://timer.cistrome.org/) was employed to systematically investigate the molecular characteristics of tumour-immune interactions. The TIMER algorithm estimates the abundance of six immune infiltrates, including B cells, CD8(+) T cells, CD4(+) T cells, macrophages, neutrophils, and dendritic cells 20. The relationship between the levels of PIK3R4 expression and immune infiltration was investigated in this study via the TIMER database.

Based on the Estimation of STromal and Immune cells in MAlignant Tumour tissues using Expression data (ESTIMATE) algorithm 21, the immune and stromal scores in the tumour microenvironment (TME) were obtained using the R package estimate.

The immunological characteristics of the TME in DLBCL include the expression of immune checkpoint genes. We first collected information on 60 immune checkpoint genes, including immune inhibitors and stimulators, from the study of Thorsson 22. The correlations between PIK3R4 expression and immune checkpoint molecule expression were calculated by Pearson's method.

### Statistical analysis

All statistical analyses were conducted using SPSS, version 20.0 (SPSS Inc., Chicago, USA) and GraphPad Prism, version 8.0 (GraphPad Software Inc.) software. The difference between two groups was analysed with Student's t test. A receiver operating characteristic (ROC) curve was constructed to analyse the effectiveness of target gene expression in distinguishing tumour and benign samples. Area under the ROC curve (AUC) values were used to evaluate the diagnostic value. An AUC value of ROC over 0.50 was considered statistically significant. P < 0.05 was considered to indicate statistical significance.

## Results

### Differential Expression of PIK3R4 in Multiple Cancer Types

**Figure 2:**
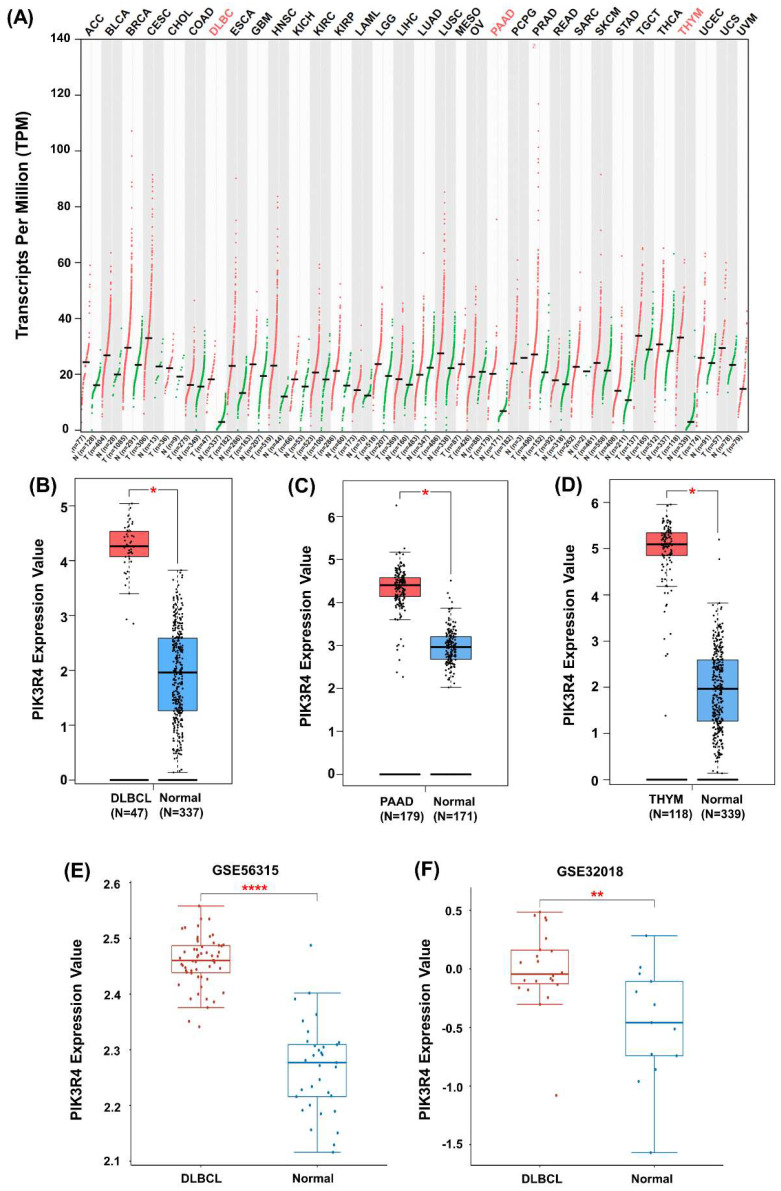
**Increased Expression of PIK3R4 in DLBCL Samples.** (A) The PIK3R4 level was increased in 3 types of tumour tissues, including DLBCL tissues, relative to normal tissues as determined through GEPIA2 analyses. (B-D) The expression of PIK3R4 were determined by GEPIA2 between DLBCL, PAAD and THYM samples, compared to normal samples. (E) The expression level of PIK3R4 in DLBCL samples and normal samples in the GSE56315 dataset. (F) The expression of PIK3R4 in DLBCL samples and normal samples in the GSE32018 dataset. *P < 0.05, **P < 0.01, ****P < 0.0001.

The differential expression of PIK3R4 in tumour samples and normal samples from all TCGA cancer types was observed (Figure 2A), the results indicated that PIK3R4 is significantly up-regulated in DLBCL, PAAD, and THYM compared to their corresponding normal tissues (Figures 2B-D). The GEO database also showed that the PIK3R4 level was significantly elevated in DLBCL tissues compared with normal tissues in the GSE56315 and GSE32018 datasets (Figure 2E-F).

### Increased Expression and Diagnostic Value of PIK3R4 in DLBCL

**Figure 3:**
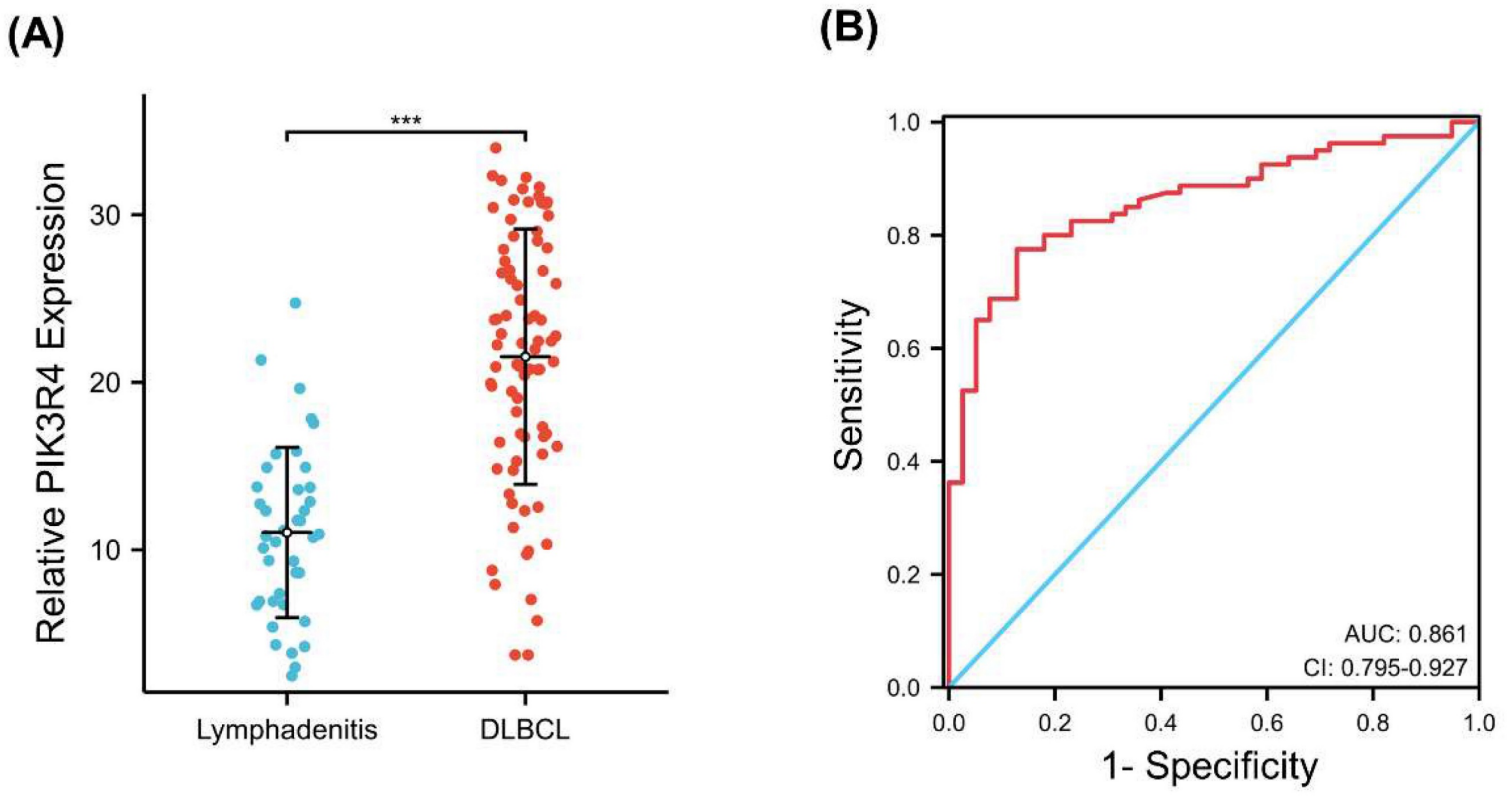
**The Diagnostic Value of PIK3R4 in DLBCL.** (A) The PIK3R4 expression in DLBCL patient samples and benign lymphadenitis tissue samples was compared. ***P < 0.001. (B) ROC curve analysis showing the performance of PIK3R4 expression in discriminating between DLBCL tissues and benign tissues. Red represents sensitive curves. The X-axis represents the false positive rate and is shown as '1-Specificity'. The Y-axis indicates the true positive rate and is shown as 'sensitivity'.

Our qRT‒PCR results revealed that the mRNA level of PIK3R4 in DLBCL tissues was significantly higher than that in benign lymphadenitis tissues (P<0.001). The median expression value for PIK3R4 in tumour tissues was 21.52 (range 3.72-33.99), while the median level of PIK3R4 in benign lymphadenitis tissues was 11.03 (range 2.47-24.73) (Figure 3A). A ROC curve was constructed to determine whether PIK3R4 could be used as a diagnostic biomarker for DLBCL. The AUC value was 0.861 (P<0.001, Figure 3B).

### The Prognostic Role of PIK3R4 expression in DLBCL Patients

**Figure 4:**
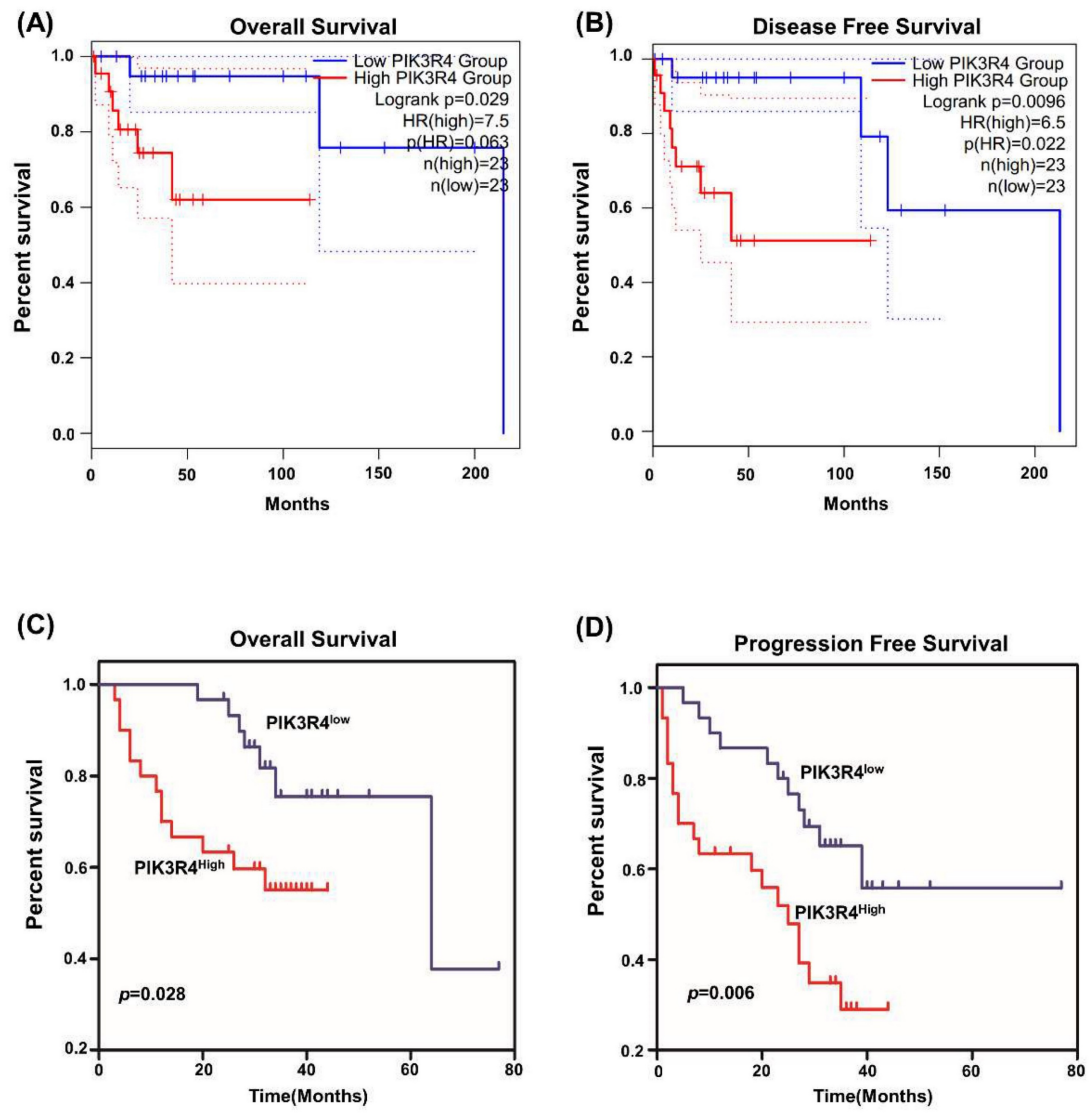
**The Prognostic Role of PIK3R4 Expression in DLBCL Patients.** (A) The relationship between PIK3R4 expression and OS of DLBCL patients according to GEPIA2. (B) The relationship between PIK3R4 expression and DFS of DLBCL patients according to GEPIA2. (C-D) Kaplan-Meier analysis of OS and PFS of DLBCL patients grouped according to PIK3R4 mRNA expression levels. Up-regulation of PIK3R4 was correlated with poor OS and PFS in DLBCL patients.

We explored the influence of PIK3R4 levels on the survival of DLBCL patients. Kaplan‒Meier curves revealed that high PIK3R4 levels were significantly associated with worse OS and DFS in TCGA datasets (Figures 4A-B). Then the relationship between PIK3R4 mRNA levels and DLBCL patients prognosis was analyzed in the 80 DLBCL patients whose PIK3R4 levels were determined by qRT-PCR. The results indicated that the OS of DLBCL patients with high PIK3R4 expression was significantly worse than that of DLBCL patients with low PIK3R4 levels (Figure 4C, P<0.05). Similarly, the PFS of the high-PIK3R4 group was significantly shorter than that of the low-PIK3R4 group (Figure 4D, P<0.01). According to these data analyses, high PIK3R4 levels could predict a poor prognosis in DLBCL patients.

### Functional and Pathway Analyses of PIK3R4 in DLBCL

**Figure 5:**
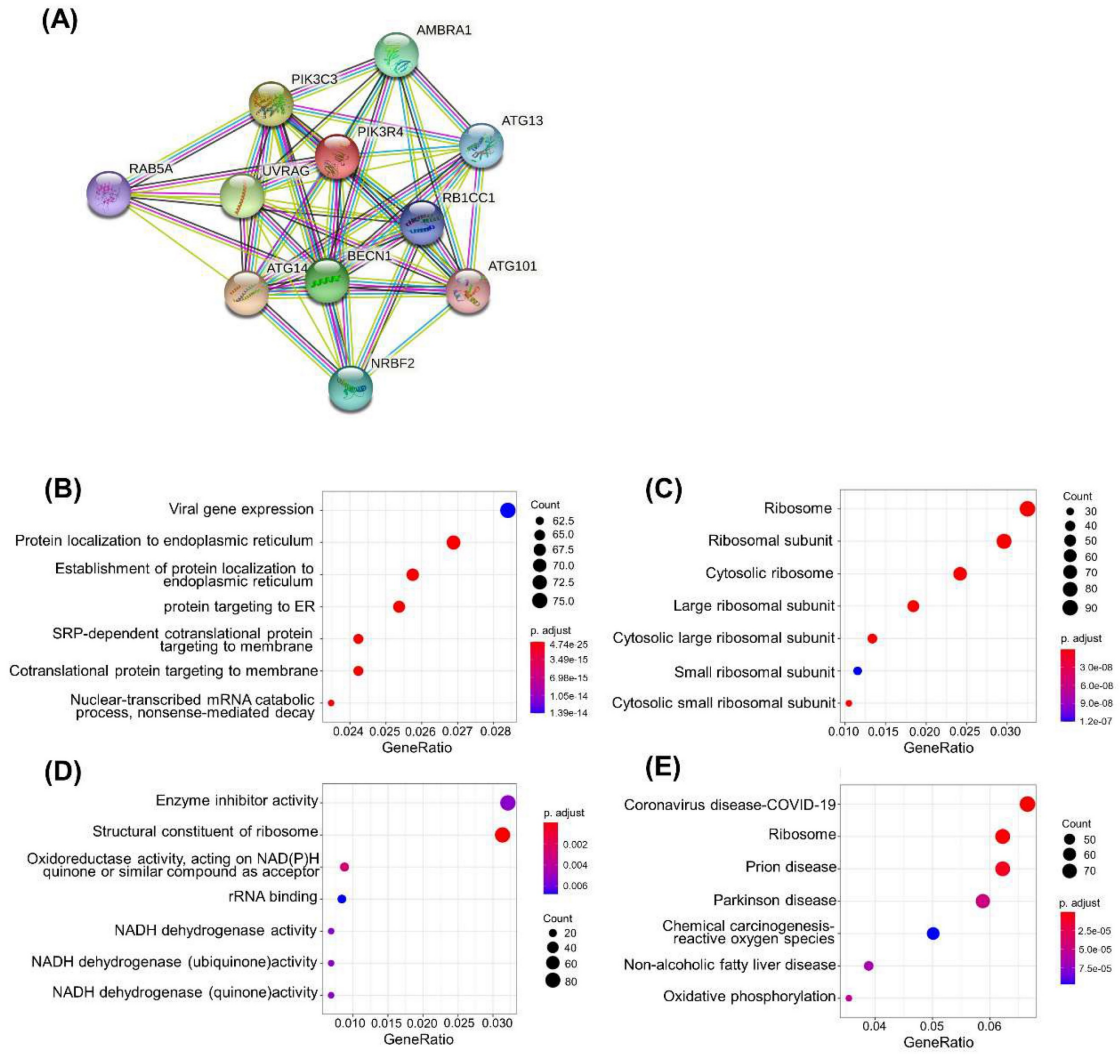
**Functional and Pathway Analyses of PIK3R4 in DLBCL.** (A) PPI network based on PIK3R4 expression. GO enrichment analysis results of biological process (B), cellular component (C) and molecular function (D). (E) Top seven enriched pathways in KEGG analysis.

As the PIK3R4 mRNA level acts as a valuable prognostic biomarker for DLBCL. PPI network analysis with the online STRING website was carried out to explore the potential relationships between PIK3R4 and other genes in DLBCL (Figure 5A). The results showed that PIK3R4 is closely related to autophagy-associated genes such as BECN1, ATG14, RB1CC1, and PIK3C3.

To explore the potential pathways mediated by PIK3R4 in DLBCL, GO and KEGG pathway enrichment analyses were performed. In GO analysis, we filtered the top seven cellular component (CC), biological process (BP), and molecular function (MF) terms based on the following criteria: p value < 0.01 and FDR < 0.05. PIK3R4-related DEGs were primarily enriched in viral gene expression and protein localization to the endoplasmic reticulum in the BP category. The enriched MFs consisted of enzyme inhibitor activity and structural constituent of ribosome. The enriched CCs were mainly related to the ribosome, ribosomal subunit, etc. (Figures 5B-D). The KEGG pathway analysis showed that COVID-19 was the most significantly enriched pathway, and ribosome ranked second (Figure 5E).

### GSEA of PIK3R4 in DLBCL

**Figure 6:**
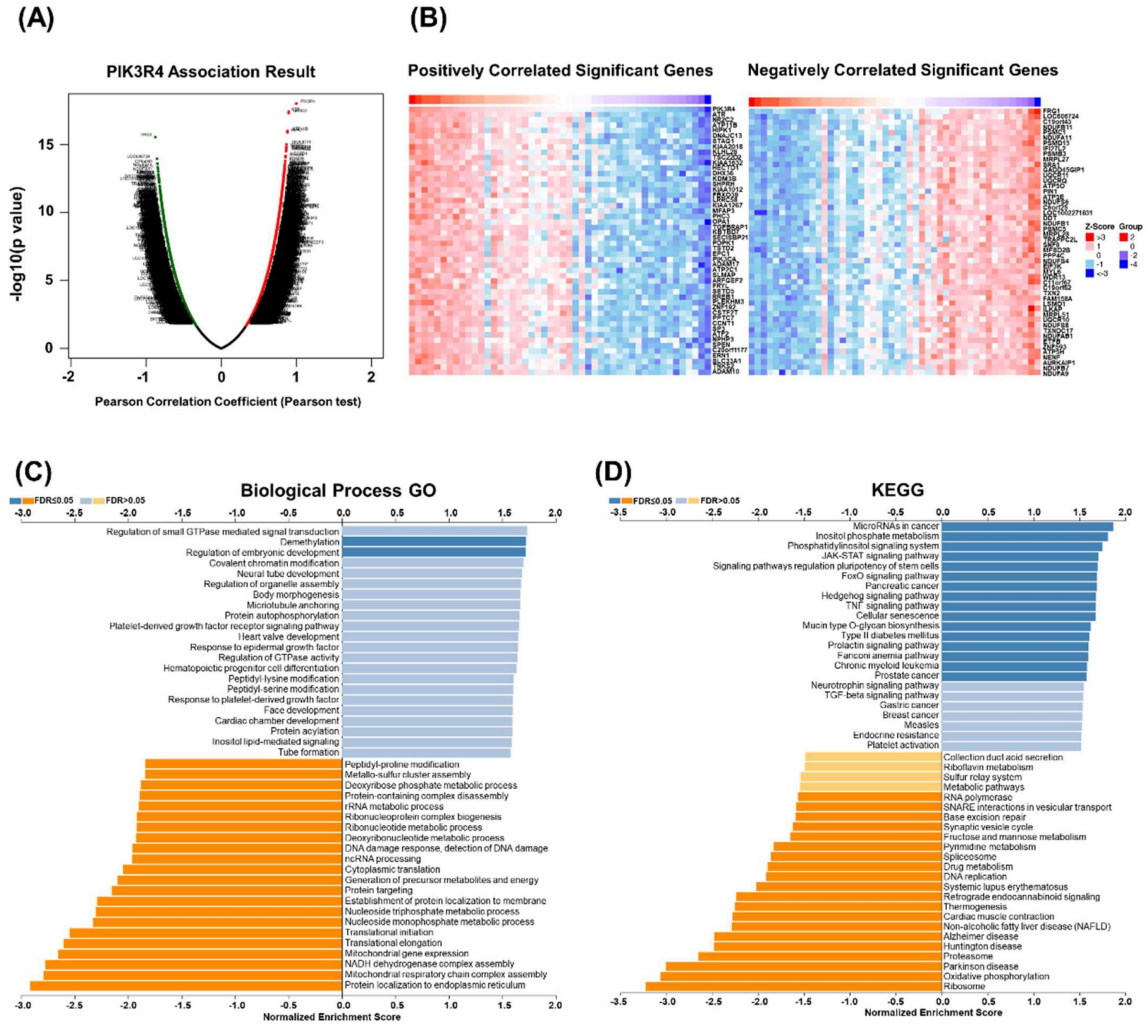
** Gene Set Enrichment Analysis (GSEA) of PIK3R4-Related Genes in DLBCL.** (A) Volcano plot of all differential expressed genes. (B) Genes co-expressed with PIK3R4 in DLBCL. (C-D) Significantly enriched Biological Processes and KEGG pathways of PIK3R4-related genes in DLBCL based on GSEA annotations (LinkedOmics).

To explore the function and potential mechanism of PIK3R4 in DLBCL, correlation analysis between PIK3R4 and various genes was performed via LinkedOmics. The top 50 positively and negatively significantly correlated genes are shown in Figure 6A-B. GO analysis in biological process by GSEA indicated that PIK3R4 co-expressed genes mainly participated in demethylation, ribosomal RNA (rRNA) metabolic process, DNA damage response, mitochondrial gene expression, and nucleoside metabolic process (Figure 6C). KEGG pathway analysis showed that PIK3R4 co-expressed genes were enriched in ribosome, oxidative phosphorylation, proteasome, and cellular senescence (Figure 6D). Ribosomes play diverse roles in the host immune response by boosting immune signaling or facilitating pathogen production under different circumstances 23, 24. Ribosomal proteins show spontaneous immunogenicity in some malignancies including breast and prostate cancer, demonstrating that ribosomal proteins might be a useful immunological target in cancer patients 25, 26. This association between PIK3R4 expression and ribosomes made us interested in its role in immunity in DLBCL.

### Associations between PIK3R4 Expression and Tumour Immune Infiltration, Tumour Microenvironment Features and Somatic Copy Number Alteration in DLBCL

**Figure 7:**
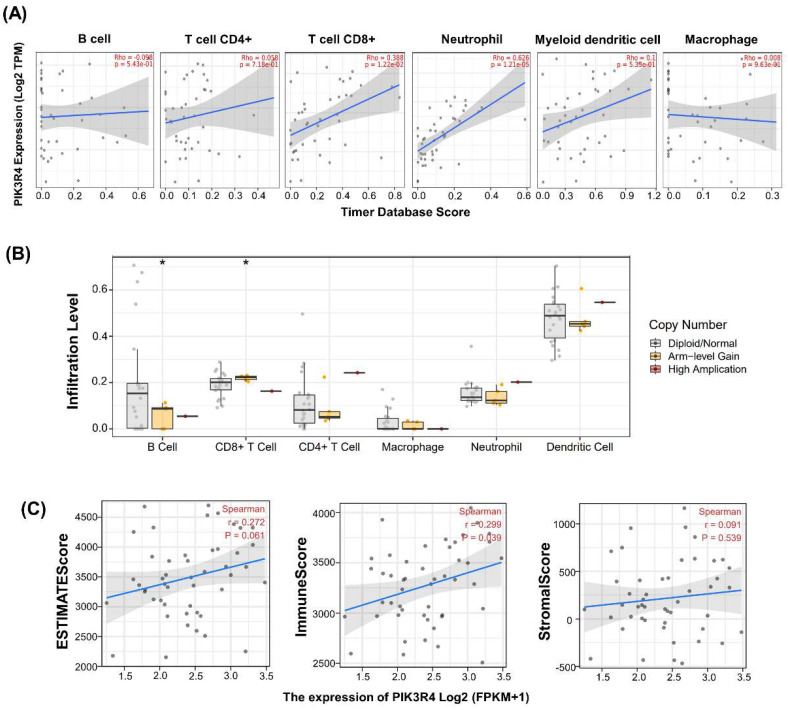
** Associations Between PIK3R4 Expression and Tumour Immune Infiltration, Tumour Microenvironment Features and SCNA in DLBCL.** (A) Associations between PIK3R4 expression and the infiltration abundances of selected immune cells in DLBCL through the TIMER database. (B) Associations between PIK3R4 expression and SCNA in DLBCL from the TCGA dataset. *P < 0.05. (C) Associations between PIK3R4 expression and tumour microenvironment features in DLBCL from the TCGA dataset are shown in a linear graph.

**Figure 8:**
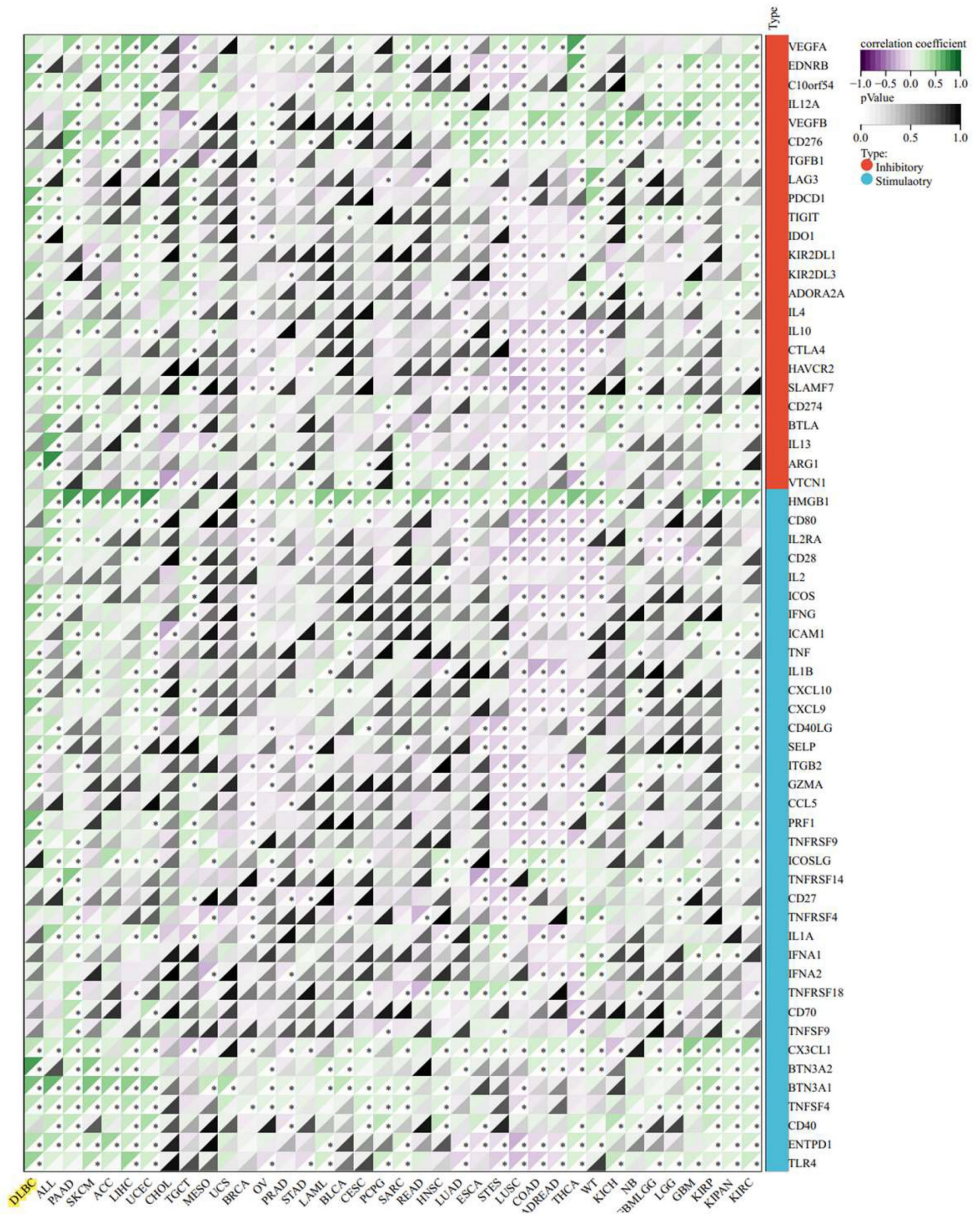
** Correlations Between PIK3R4 Expression and Immune Checkpoint Molecules in DLBCL from the TCGA Dataset.** *P < 0.05.

In consideration of the clinical successes of immunotherapy for cancer treatment, clarifying the interaction mechanism between malignant cells and the host immune system is necessary. We investigated the relationship between PIK3R4 expression and immune cell infiltration in the TCGA-DLBC cohort via TIMER database analysis. In DLBCL patients, high PIK3R4 expression was markedly related to CD8(+) T-cell and neutrophil infiltration (P < 0.05, Figure 7A), suggesting a significant positive correlation between the PIK3R4 level and the immune infiltration level. Then, the somatic copy number alteration (SCNA) module provided the correlations between tumour immune infiltration levels among DLBCL and different SCNAs for PIK3R4 by the Wilcoxon rank-sum test (Figure 7B). The correlation analysis showed that the PIK3R4 level was significantly associated with the ImmuneScore in DLBCL with Spearman's method (P < 0.05), whereas it was not linked to the ESTIMATEScore or StromalScore (Figure 7C). These findings suggest that PIK3R4 affects patient survival by interacting with immune infiltration in DLBCL. Finally, the correlations between PIK3R4 expression and immune checkpoint molecule expression were calculated, and the results showed that PIK3R4 mRNA expression was significantly associated with BTN3A2, BTN3A1, PRF1, CXCL9, PDCD1, and TIGIT expression in DLBCL (Figure 8, all P < 0.05).

## Discussion

As a prevalent primary malignant tumour of the lymphoid system, the heterogeneity of DLBCL remains a challenge for clinicians in terms of the development of individualized treatments. Preclinical and clinical immunotherapies, including immune checkpoint inhibitors and CAR-T therapies, have been used for DLBCL treatment. Unfortunately, more than 40% of DLBCL cases eventually progress to therapy-resistant relapsed or refractory disease. A deeper understanding of the genetic landscape and molecular features will contribute to identifying high-risk subsets of DLBCL patients with poor responses to chemotherapy/immunotherapy 27. It is necessary to determine more potential immune-related prognostic biomarkers for predicting the response to immunotherapies in DLBCL.

As the response of tumour cells to external stress, autophagy is a double-edged sword for lymphoma. Many lymphoma-related genes, such as BECN1, BCL-2 family molecules and TP53, are involved in autophagy. DLBCL patients with decreased BCL-2 levels have been identified to have increased BECN1 expression, and these phenotypes correlate with a favourable clinical outcome 28. As a scaffold protein of the CUL4B-RING E3 ubiquitin ligase complex, Cullin4B (CUL4B) can regulate autophagy through JNK signalling. Besides, CUL4B deletion-induced cell proliferation inhibition may be attributed to the blocking of the pro-survival ability mediated by autophagy, demonstrating the important role of autophagy in DLBCL progression 29.

As a novel member of the autophagy pathway, PIK3R4 plays a crucial role in several malignant tumours progression, but its role and molecular mechanism in DLBCL are unclear. Here, we first analyzed the change in PIK3R4 expression in DLBCL tissues using bioinformatics analysis, and PIK3R4 was found to be expressed differently in diverse cancer tissues. Particularly, the level of PIK3R4 in DLBCL samples was significantly higher than that in benign tissues. ROC curve analysis showed that PIK3R4 has potential diagnostic value for DLBCL. TCGA database analysis revealed that high PIK3R4 expression was associated with a higher immune score and worse prognosis. GO analysis revealed that PIK3R4-related genes in DLBCL are enriched in rRNA metabolic process, DNA damage response, mitochondrial gene expression, and nucleoside metabolic process. KEGG analysis showed that PIK3R4-related genes in DLBCL are enriched in ribosome, oxidative phosphorylation, proteasome, and cellular senescence. More importantly, Kaplan-Meier analysis indicated that DLBCL patients with high PIK3R4 expression had worse OS and PFS than patients with low PIK3R4 expression. These results indicated that PIK3R4 was involved in DLBCL progression and immune-related pathways in the DLBCL microenvironment.

The dynamic characteristics of tumour immune infiltration, the tumour microenvironment, immune checkpoint molecules and immune cell pathways are vital in regulating the response to immunotherapy and have been verified to serve as biomarkers to evaluate tumour cell responses to immunotherapy and affect patient prognosis 30, 31. The expression of immune checkpoint proteins is related to sensitivity to immune checkpoint blockade 32. Our results revealed that PIK3R4 is a potential indicator of immunotherapy response in DLBCL and has certain clinical guiding significance. First, the PIK3R4 expression in DLBCL is positively correlated with immune cell content in the cancer microenvironment. Second, PIK3R4 mRNA expression is markedly correlated with CD8(+) T-cell and neutrophil infiltration. Third, PIK3R4 mRNA expression is significantly associated with the levels of immune checkpoint molecules, such as BTN3A2, BTN3A1, PRF1, CXCL9, PDCD1, and TIGIT, in DLBCL.

There are still some limitations in our study. Differences in sample sizes among multiple databases might cause some bias, and more efforts should be made to investigate the molecular mechanism of PIK3R4 during DLBCL progression. Further studies with larger sample sizes and experiments in vivo and in vitro are required to verify our findings.

Together, our data suggest that PIK3R4 functions as a novel regulator of immune cell infiltration within the DLBCL tumour microenvironment and is a valuable potential prognostic biomarker for DLBCL patients.
